# Cylindrical Shell Vibration Gyroscope Excited and Detected by High-Temperature-Sintered Piezoelectric Ceramic Electrodes

**DOI:** 10.3390/s20215972

**Published:** 2020-10-22

**Authors:** Tianliang Qu, Guanqing Zhou, Xiaoming Xue, Junhua Teng

**Affiliations:** 1 School of Information Science and Technology, Dalian Maritime University, Dalian 116026, China; xxm1633588825@dlmu.edu.cn (X.X.); tengjunhua@dlmu.edu.cn (J.T.); 2 College of Advanced Interdisciplinary Studies, National University of Defense Technology, Changsha 410073, China; zhouguanqing13@nudt.edu.cn

**Keywords:** cylindrical shell piezoelectric vibration gyroscope, quartz resonator, piezoelectric ceramic electrode, Q factor, piezoelectric excitation and detection, high-temperature sintering

## Abstract

A cylindrical shell piezoelectric vibration gyroscope is a kind of Coriolis vibration gyroscope. Its core components are the cylindrical quartz resonator (CQR) and the piezoelectric ceramic electrodes (PCEs). In order to develop a high-precision Cylindrical shell piezoelectric vibration gyroscope, it is very important to reduce the influence of the PCEs and obtain a high-quality-factor CQR. To achieve this goal, a novel high-temperature sintering method is proposed to combine the CQR and the PCEs, and the corresponding sintered resonators are fabricated. After sintering, results of the acoustic excitation experiment and piezoelectric excitation experiment are tested, and the influence of the sintered PCEs on the CQR is determined. A complete gyroscope is obtained by vacuum packaging the sintered resonator. Through the open-loop and closed-loop tests, the performance parameters of gyroscope are obtained. The feasibility of the high-temperature sintering method is proved by experiments.

## 1. Introduction

A cylindrical resonator gyroscope (CRG) can detect the angular velocity of the inertial space object by the standing wave precession. Compared with traditional gyroscopes, such as the mechanical rotor gyroscope and the liquid floated gyroscope, CRG has no rotating parts and avoids mechanical friction, so it has the advantages of low energy consumption, fast start-up, and high stability [1]. CRG has great advantages in measurement accuracy, operation reliability, working life, production cost, etc., and has broad application prospects in navigation of mobile platform, autopilots for guidance and control of aircraft, missiles, ships, and land vehicles, etc. [2,3]. The cylindrical resonator is the core component of CRG, which determines the performance of the CRG [4]. Resonators are usually made of metal, ceramics, quartz, or other materials [2,3,4,5,6]. Among them, the quartz has excellent isotropy and is an ideal resonator material. By improving the processing technology, the quality factor (Q factor) of cylindrical quartz resonator (CQR) can be better than 8 × 10^5^ in low vacuum [7].

There are two common ways of excitation and detection for shell vibration gyroscope: piezoelectric excitation and detection, and electrostatic excitation and detection [8]. Due to the advantages of the large driving force, high sensitivity, simple structure, reliability of driving, and detecting performance [9], excitation and detection by piezoelectric ceramics have been widely used. Quality factor (Q) is the key parameter for CRG. For piezo-electric CRG, why is the precision relatively lower? Because the quality factor of the silica resonator glued with piezo-electric electrodes is lower, and the gluing method is bad for Q factor which will introduce, large interface loss. The combination of the piezoelectric ceramic electrodes (PCEs) and the CQR will lead to a sharp decrease in the overall Q factor. In addition, the epoxy glue used to bond the PCEs to the CQR will also aggravate the decline of the Q factor and organics in the epoxy glue will continue to deflate in the vacuum environment, breaking the working environment of the CQR [10]. Based on the above problems, our group once proposed a scheme of piezoelectric film excitation and detection, in which piezoelectric films were uniformly coated at the bottom of the resonator. As the thickness of the piezoelectric film was only a few microns, the influence on the Q factor could be ignored, and the Q factor could be as high as 2.89 × 10^6^ [11]. Without the introduction of the organic glue, it is helpful to maintain the working environment. However, there were other problems, such as the poor consistency of the piezoelectric films and sometimes large leakage current. In [4], they researched the Q factor of the trimmed metal resonator for vibratory cupped gyroscopes, however they did not consider the effect of piezoelectric electrodes.

Based on the problems existing in the method of bonding the PCEs to the CQR by epoxy glue, the high-temperature sintering method is innovatively proposed and the Q factor of the silica resonator could indeed be improved compared with the gluing method. The corresponding design, experiment, and analysis are carried out. The main contents of this paper are as follows: in Section 2, using the COMSOL software, the piezoelectric excitation and detection of two kinds of CQRs are simulated and compared. In Section 3, a high-temperature sintering method is proposed and the corresponding sintered resonators are fabricated. In Section 4, the results of piezoelectric excitation and acoustic excitation are tested. The resonator is vacuum packaged to get the whole gyroscope. The gyroscope is tested on the open-loop test system based on the LabVIEW platform, and the appropriate vibration parameters are obtained. The gyroscope performance parameters are obtained on the closed-loop test system based on the FPGA (Field Programmable Gate Array);platform. In Section 5, experimental results are compared and discussed, and we come to the conclusion that it is feasible to combine the PCEs with the CQR by the high-temperature sintering method, but the scheme is still in the initial stage. To obtain a high precision cylindrical shell piezoelectric vibration gyroscope, several improvement methods are summarized. This work paves a way for a high-precision, small-size, and low-cost CVG in the future.

## 2. Simulation

**Figure 1:**
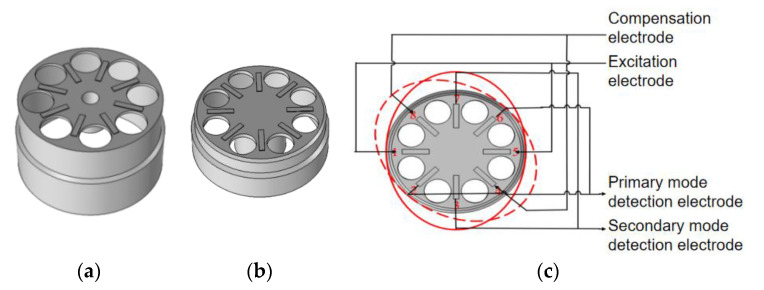
(**a**) Cylindrical resonator HCVG with the piezoelectric electrode; (**b**) cylindrical resonator LCVG with the piezoelectric electrode; (**c**) the distribution of piezoelectric electrodes on the resonator.

The vibration and modal analysis of the resonator involve complex shell dynamics theory, and it is difficult to get an accurate numerical solution through theoretical calculation [12]. To further verify the correctness of the theory, the cylindrical resonator models combined with PCEs were built in the COMSOL software. We had two kinds of resonators in hand, the models are shown in Figure 1a,b. One was the optimized model based on the cylindrical resonator of the Innalabs company and named HCVG [13] (high CVG with diameter D = 12.5 mm, height h = 20 mm, thickness t = 1.0 mm); the other was the model designed by the research group and named LCVG (low CVG with diameter D = 12.5 mm, height h = 10 mm, thickness t = 1.5 mm). The PCE distribution is shown in Figure 1c.

To compare the simulation results of the two models conveniently, the cylindrical resonator materials of two models were uniformly set as fused quartz, and the vibration parameters of the materials were the same; the PCE materials were uniformly set as PZT-5H, and the piezoelectric parameters of the materials were the same.

**Figure 2:**
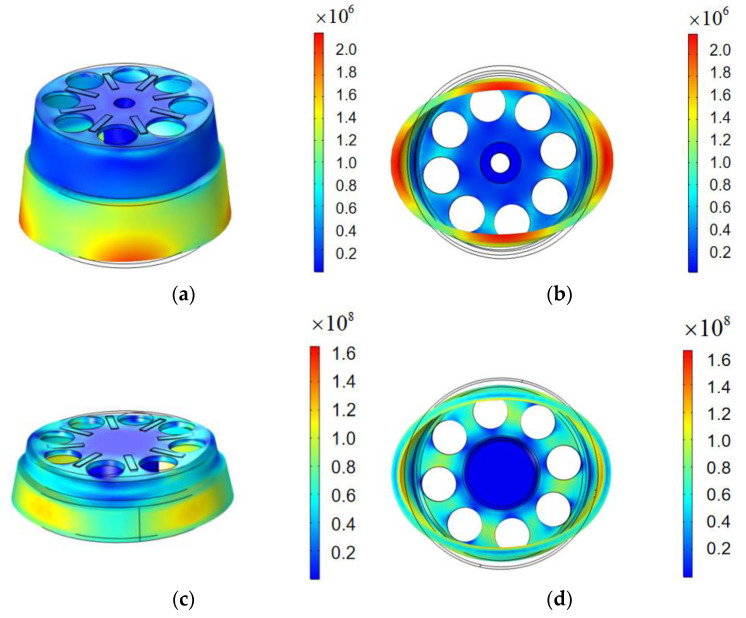
(**a**) Front view of HCVG piezoelectric excitation simulation; (**b**) top view of HCVG piezoelectric excitation simulation; (**c**) front view of LCVG piezoelectric excitation simulation; (**d**) top view of LCVG piezoelectric excitation simulation.

Through the COMSOL software, the eigenfrequencies of two models were determined as 4962.9 Hz (HCVG) and 8019.4 Hz (LCVG). The driving signal was applied with the eigenfrequency on the excitation electrode, making the resonator vibrate stably in the four-wave belly vibration [7] mode. The vibration results are shown in Figure 2.

A point on the bottom edge of the resonator was selected, corresponding to the excitation electrode, and the vibration rate and displacement in the Z-direction measured under the steady excitation state. The voltages on three pairs of electrodes at ±45° and 90° with the excitation electrode were detected to inspect the sensitivity and efficiency of the piezoelectric detection.

**Figure 3:**
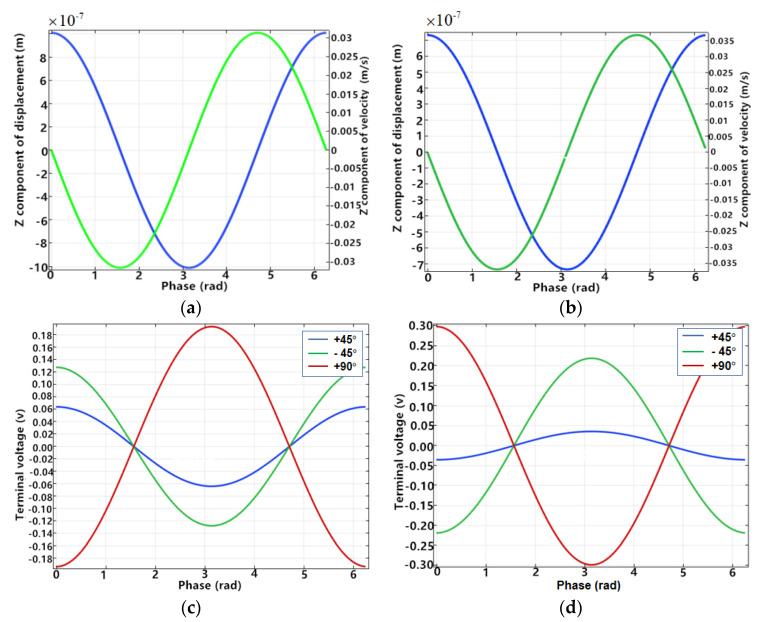
(**a**) Detected displacement (blue) and velocity (green) of HCVG piezoelectric excitation in Z direction; (**b**) detected displacement (blue) and velocity (green) of LCVG piezoelectric excitation in Z direction; (**c**) detected voltages of HCVG piezoelectric detection in Z direction; (**d**) detected voltages of LCVG piezoelectric detection in Z direction.

As shown in Figure 3a,b, the blue lines are the z-component of the displacement, and the green lines represent the z-component of the rate. Driven by the same amplitude signal, the displacement and the peak rate of HCVG reached 1000 nm and 0.03 m/s, respectively, while those of LCVG were 800 nm and 0.035 m/s, respectively. The displacement of HCVG was slightly larger and the rate of LCVG was slightly larger, but they were in the same order of magnitude generally.

As shown in Figure 3c,d, the peak values of detected voltages at the positions of 90°, +45°, and −45° on the HCVG were 0.19303 V, 0.06395 V, and 0.12758 V, respectively, and those of the LCVG were 0.29824 V, 0.03549 V, and 0.2188 V, respectively. As far as the ideal resonator is concerned, the detected voltages at ±45° should be the same. However, due to the existence of the positioning hole at the bottom of the CQR, it was impossible to ensure that the position of the excitation electrode and the primary axis were completely coincident, so the detected voltages at the position of ±45° were not equal. Compared with HCVG, the detected voltages of LCVG were larger. In terms of structure, LCVG is more suitable for piezoelectric excitation and detection.

## 3. Fabrication and Characterization

### 3.1. Fabrication

#### 3.1.1. Front-End Processing of the Resonator

**Figure 4:**
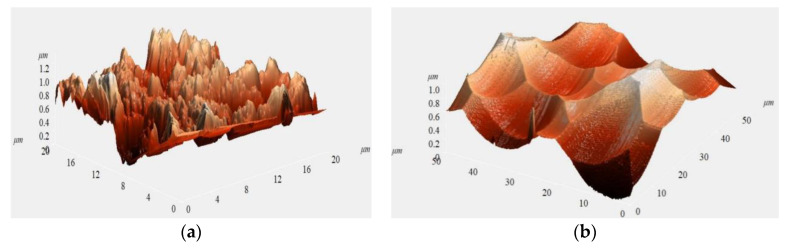
(**a**) Surface topography of the initially machined resonator; (**b**) surface topography of the resonator after chemical polishing.

Based on the structures of HCVG and LCVG, two bare CQRs named HCVG#01 and LCVG#01 were fabricated [14]. As shown in Figure 4a, there was a structural damage layer on the resonator surface after initial processing. There were still some unreleased internal stresses in the quartz material, which had a great influence on the vibration characteristics. Therefore, the initially machined resonator was annealed at 1050 °C for 12 h in an electric vacuum furnace to release the internal stress. The resonator was placed in the chemical polishing solution to remove the surface damage layer and improve its surface morphology [7]. Figure 4b shows the surface topography of the resonator after chemical polishing.

Due to the limitation of processing technology, there was a certain structural asymmetry in the resonator, which would lead to a frequency split. Large frequency split was not conducive to the improvement of gyroscope precision, so the chemical modification method was used to remove the tiny mass from the four points corresponding to the low-frequency axis to reduce frequency split.

After the high-temperature annealing, chemical polishing, and chemical modification, HCVG#01 and LCVG#01 were improved.

#### 3.1.2. Research on the Combination Scheme of the Resonator and Piezoelectric Ceramic Electrodes

Through the analysis of the existing scheme of epoxy glue, it was found that the local mass, stiffness, and damping characteristics of the CQR will be changed when the PCEs are pasted on the resonator with epoxy glue, resulting in a sharp decline in the Q factor and seriously hampering the high-precision development of the cylindrical shell piezoelectric vibration gyroscope. Based on the above problems, a scheme of high-temperature sintering was put forward and the corresponding researches were carried out.

##### Precise Positioning of the Piezoelectric Ceramic Electrodes

The asymmetry of the electrode position will increase the frequency split and reduce the Q factor. Therefore, the precise positioning of the PCEs has always been an important problem in the research of the piezoelectric shell vibration gyroscope.

**Figure 5:**
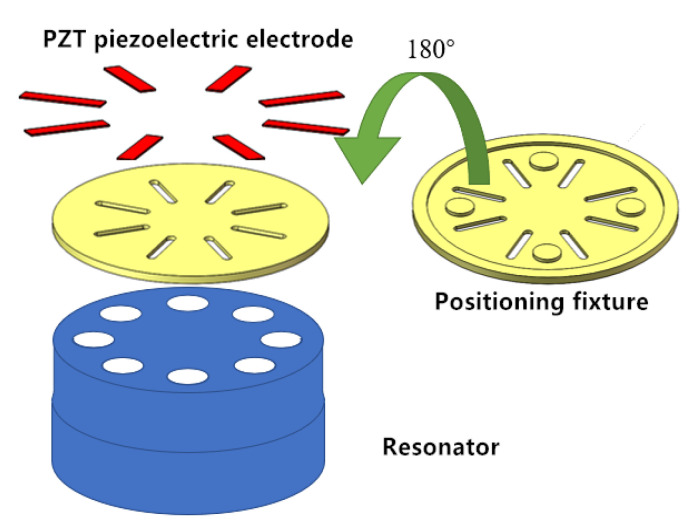
Piezoelectric electrode positioning and installation fixture.

For the consideration of the processing technology, the positioning fixture was designed. As shown in Figure 5, there were eight symmetrical voids that were the same size as the PCEs. The precise position of the PCEs was determined by matching the four positioning columns on the fixture with the symmetrical circular holes at the bottom of the resonator. The PZT piezoelectric electrodes were installed at the bottom of the CQR through the voids. The advantage of this method is that the fixture is easy to process, it can ensure high symmetry precision, it is easy to operate, and does not disturb the quality of the resonator.

##### Bonding of the Piezoelectric Ceramic Electrodes

In the field of piezoelectric ceramic processing, the silver powder is often sintered to the PZT surface by high-temperature sintering to form the thin silver layer electrode. Inspired by the above processing, a novel high-temperature sintering method was proposed to sinter the PCEs onto the CQR. The binder used in this method is turpentine with silver powder and glass powder mixed evenly. Turpentine volatilizes when heated at a high temperature, which would not have a negative impact. Glass powder plays the role of adhesion and combustion aid, ensuring the strength of high-temperature sintering. Silver powder is beneficial to the conductivity of the lower surface of the electrode. To coat the binder uniformly, the Screen Printing Technique in the field of circuit board printing was taken as a reference. The printing screen was made, in which eight long strip patterns corresponded to eight PCEs, and the circle in the middle was connected with eight surrounding electrodes as a common ground electrode. After coating the upper layer of the screen, the uniform patterns would be left under the screen mold.

**Figure 6:**
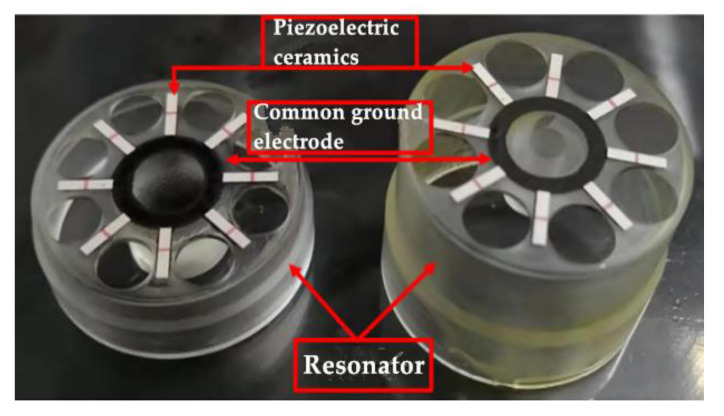
Resonators pasted with piezoelectric ceramic electrodes (the yellow substance on HCVG#01 is the residual wax, which will volatilize at the high temperature and not affect the quality of the resonator).

For this new scheme, we firstly explored it on a quartz flat plate, which was consistent with the bottom of the CQR. The quartz plate was fixed on the support column, and the uniform electrode patterns were obtained by the screen printing. After several times of printing exploration based on the quartz flat plate, aiming at the problems of slight leakage of binder, slight shaking of quartz flat plate, improper bonding time, etc., the appropriate printing conditions were determined. Then, binder printing and electrode bonding were carried out for HCVG#01 and LCVG#01, respectively. Results are shown in Figure 6.

##### High-Temperature Sintering of the Piezoelectric Ceramic Electrodes

The PCEs pasted by turpentine binder have no mechanical strength, so they need to be sintered onto the CQR. The optimum sintering conditions were determined by many times of exploration based on the quartz plate pasted with PCEs by turpentine binder. After sintering, PCEs on the quartz flat plate were applied destructive force until they were crushed, and there was no electrode fracture. The above steps were repeated for the PCEs pasted by epoxy glue, during which one of electrodes broke from the middle. It was proved that the PCEs and quartz plate were well combined after the high-temperature sintering.

HCVG#01 and LCVG#01 that were combined with PCEs by turpentine binder were put into the heating furnace, and heated according to the optimum sintering conditions obtained from the quartz flat plate exploration. After sintering, PCEs were distributed uniformly and symmetrically with high strength, the upper and lower surfaces were completely separated without short circuit, and the common ground electrode was well connected.

##### Polarization of the Piezoelectric Ceramic Electrodes

Due to the influence of high temperature in the sintering process, the piezoelectric and reverse piezoelectric effects of the PCEs were lost, so it was necessary to polarize the electrodes.

First of all, the CQR and the base should be assembled. Because the HCVG#01 shell was relatively high and the internal space was relatively sufficient, the “three-piece set” packaging shell was adopted and the vacuum glue was used to fix the package. After encapsulation, the upper and lower surfaces of the PCE need to be connected with the pin through the lead. To reduce the error of the welding spot, the spot welding technology was selected. The PCE was connected with the conductive pin by spot welding, and the lead was taken out from the base for the polarization. According to the size parameters and piezoelectric coefficient of the PCE, it was determined that the appropriate polarization condition was to heat the resonator to 160 °C in the methyl silicone oil bath and apply 260 V DC voltage on the upper and lower surfaces of the electrode for one hour. After polarization, the resonator needs to be cleaned. Because methyl silicone oil has strong adhesion and is insoluble in water and alcohol, gasoline was selected as the cleaning solution. The resonator was immersed in gasoline for several hours, then washed with gasoline, and finally heated in alcohol for cleaning. As the welding spot was very small and its physical strength was poor, the lead of HCVG#01 fell off twice in the polarization process and three times of welding wire were carried out in total, each time of re-spot welding needed to be repeatedly heated, in addition to the alcohol heating and boiling in the cleaning process, resulting in the fracture and falling off of a piezoelectric electrode.

Due to the short shell of LCVG#01, it was impossible to set the electrode disk inside. Its packaging structure was designed as a “two-piece set” structure of base and packaging cover. Because there was no electrode disk in the assembly structure, it was directly from the PCE to the pin lead. The spot welding machine has strict requirements for welding spots, so it is unable to form stable welding spots on pins. Therefore, the common soldering technology was adopted for LCVG#01. The resonator was cleaned according to the cleaning process mentioned above. The experience and lessons of HCVG#01 were learned in the treatment of LCVG#01, and the cleaning was more careful. During cleaning, the immersion time of gasoline was prolonged, the vibration cleaning was replaced by flushing, and the alcohol was properly heated, so as to ensure the integrity of the PCE.

### 3.2. Characterization

#### 3.2.1. Characterization of the Resonators after Chemical Modification

**Figure 7:**
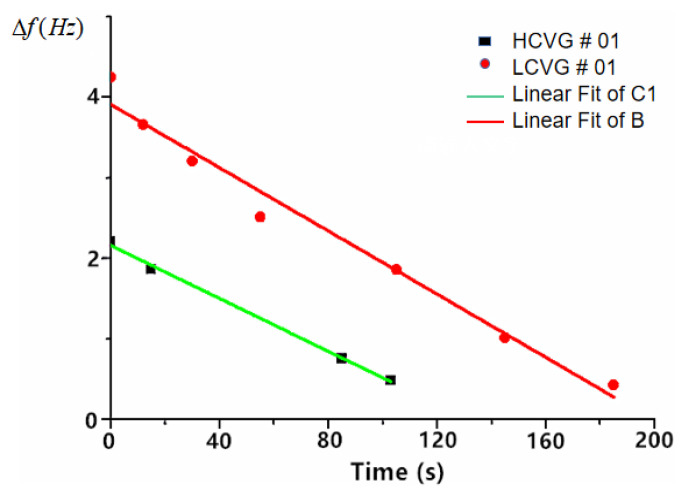
Trend of frequency split in chemical modification process.

The initial frequency split of HCVG#01 was 2.205 Hz. After three times of chemical modification in the condition described in ref [7] for 103 s, the frequency split was optimized to 0.48 Hz. The initial frequency split of LCVG#01 was 4.248 Hz, after six times of chemical modification for 185 s, the frequency split was optimized to 0.427 Hz. As shown in Figure 7. The frequency split and the modification time were approximately linear. The final frequency splits of the two resonators were close, which is advantageous to the comparison of subsequent experiments.

#### 3.2.2. Characterization of the Resonators after Front-End Processing

After the high-temperature annealing, chemical polishing, and chemical modification, HCVG#01 and LCVG#01 were improved. Then, the resonator was fixed on the base, and the working environment was maintained at around 10 Pa by the vacuum pump. The vibration characteristics of the resonator were tested by the Laser Doppler vibrometer [7]. Taking the acoustic wave as the excitation source [15,16], the primary axis was found by the vibration rate scanning of the antinode and node and then taken as the excitation axis. The eigenfrequency of the CQR was obtained by the frequency sweep and the signal of the eigenfrequency was used to drive the resonator vibration. After the vibration amplitude was stable, the excitation signal was cut off to make the vibration decay freely (this method could be also applied with a burst signal), then the decay time was recorded to calculate the Q factor [7].

**Figure 8:**
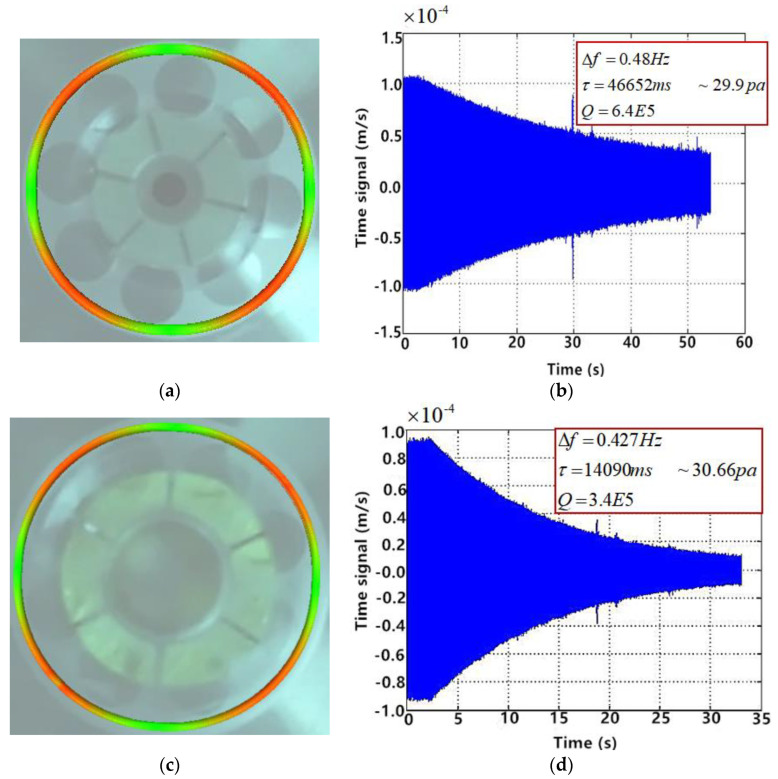
(**a**) HCVG#01 four-wave belly vibration mode diagram; (**b**) HCVG#01 vibration decay diagram; (**c**) LCVG#01 four-wave belly vibration mode diagram; (**d**) HCVG#01 vibration decay diagram.

The results are shown in Figure 8. The optimal Q factor of HCVG#01 was 6.4 × 10^5^ (30.8 Pa). The optimal Q factor of LCVG#01 was 7.9 × 10^5^ (0.021 Pa), to facilitate the comparison, the Q factor of LCVG#01 was 3.4 × 10^5^ (30.66 Pa). The frequency split of HCVG#01 was 0.48 Hz, while that of LCVG#01 was 0.427 Hz. In terms of initial quality, the Q factor of HCVG#01 was better and the frequency split of LCVG#01 was better.

## 4. Experimental Results

### 4.1. Acoustic Excitation Experiment of the Resonator after Sintering

**Figure 9:**
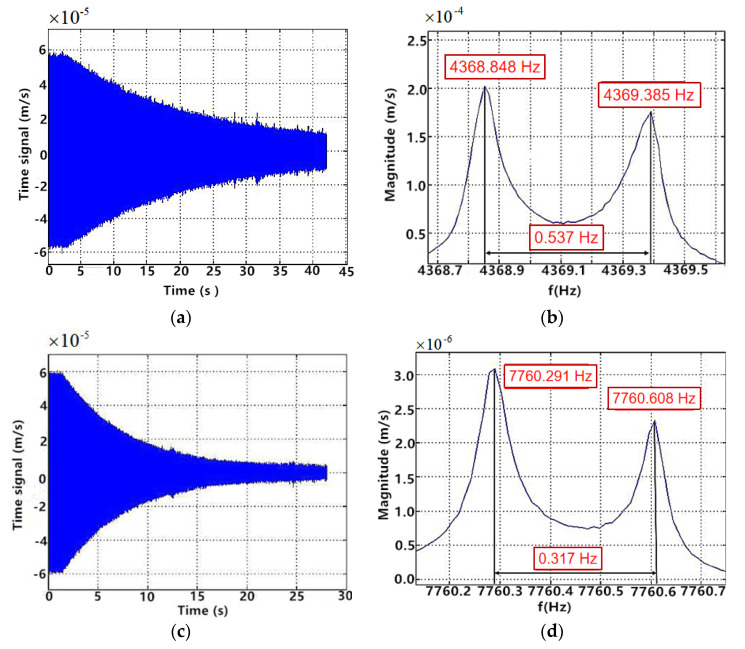
(**a**,**b**) Test results of HCVG#01 resonator after sintering; (**c**,**d**) test results of LCVG#01 resonator after sintering.

Through the high-temperature sintering, the PCEs and the CQR formed a whole. To analyze the influence of the electrodes sintering on the resonator, the vibration characteristics were obtained by the acoustic excitation experiment. The sound source used the ordinary loudspeaker, the rated voltage was 5 V, the output power was 3 W. The test environment and method were the same as those before adding electrodes. The measurement method of the frequency split in vacuum environment was the frequency sweep in the direction of the primary axis. Test results of HCVG#01 and LCVG#01 are shown in Figure 9.

To facilitate the comparative analysis, Table 1 lists the vibration parameters before and after sintering.

The amplitude of HCVG#01 in air was 3.85 nm, compared with 4.16 nm, the decrease rate was 7.45%. The amplitude of LCVG#01 in air was 3.53 nm, compared with 3.56 nm, the increase rate was 0.84%. At a vacuum of 30 Pa, the Q factors of HCVG#01 before and after sintering were 6.4 × 10^5^ (30.8 Pa) and 3.2 × 10^5^ (29.99 Pa), respectively; the Q factors of LCVG#01 before and after sintering were 3.4 × 10^5^ (29.97 Pa) and 2.2 × 10^5^ (29.5 Pa), respectively. Under the same vacuum degree and the same excitation condition, the vibration rate of HCVG#01 decreased from 0.09098 mm/s to 0.06259 mm/s, and that of LCVG#01 decreased from 0.10721 mm/s to 0.04558 mm/s. After sintering, the frequency split of HCVG#01 increased from 0.48 Hz to 0.537 Hz and that of LCVG#01 decreased from 0.427 Hz to 0.317 Hz.

### 4.2. Piezoelectric Excitation and Detection Experiment of the Resonator after Polarization

After polarization, the piezoelectric effect and reverse piezoelectric effect were restored. Therefore, the piezoelectric excitation and detection experiments of HCVG#01 and LCVG#01 were carried out. The experimental instrument was the Laser Doppler vibrometer, but the acoustic wave was not used as the excitation source. The excitation electrode was connected to the signal generator for excitation, the detection electrode was connected to the oscilloscope for detection, the common ground electrode, the oscilloscope ground terminal, and the signal generator ground terminal were connected to the unified ground, and the Laser Doppler vibrometer was used to measure vibration characteristics.

#### 4.2.1. Piezoelectric Excitation and Detection of LCVG#01

**Figure 10:**
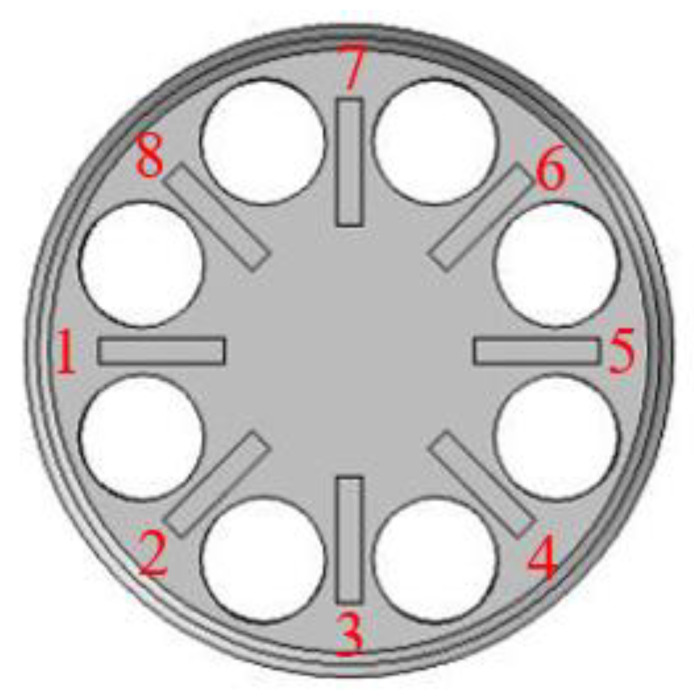
Number of piezoelectric electrode. Electrode pair 1 consists of electrode 1 and electrode 5; Electrode pair 2 consists of electrode 2 and electrode 6; Electrode pair 3 consists of electrode 3 and electrode 7; Electrode pair 4 consists of electrode 4 and electrode 8.

**Figure 11:**
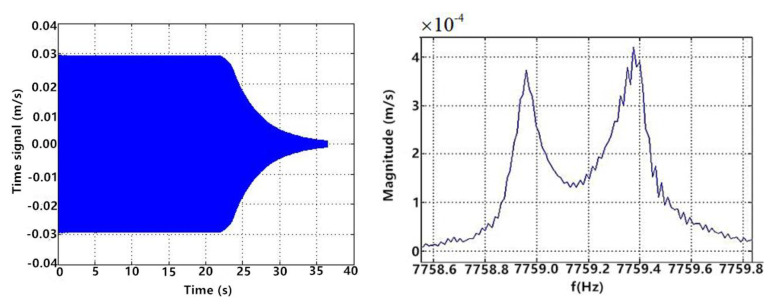
Test results of LCVG#01 when electrode pair 1 was excited.

In the vacuum environment of 0.67 pa, four pairs of electrodes were used for piezoelectric excitation successively. The electrode number is shown in Figure 10. Test results are shown in Table 2. When exciting the electrode pair 1, the result was the best and the test curve is shown in Figure 11.

In addition to exciting the resonator vibration, it is also important to use the piezoelectric electrodes to detect the vibration characteristics. The quality of the detection signal determines the signal-to-noise ratio of the gyroscope. When one of four electrode pairs was used as the excitation electrode, the oscilloscope was used to observe the other three pairs. The results are shown in Table 3.

#### 4.2.2. Piezoelectric Excitation and Detection of HCVG#01

Four pairs of PCEs were used as the excitation electrode to scan the frequency and vibration mode in the air. Only at electrode 3 could a good four-wave belly vibration mode be obtained. It could be considered that the direction of electrode 3 was the direction of the primary axis. The other three pairs of electrodes could be used as the excitation electrode to obtain the eigenfrequency, but the sweep mode was a plane, so the four-wave belly vibration mode was not obtained. In the vacuum environment of 0.023 Pa, electrode 3 was used as the excitation electrode, the Q value was tested as only 2 × 10^4^ and the frequency split was 1.17 Hz.

### 4.3. Open-Loop Test Based on LabVIEW

Because one of the PCEs of HCVG#01 broke during the polarization, the quality of HCVG#01 suffered a precipice fall. Therefore, the following experiment focused on LCVG#01.

A complete gyroscope was obtained by vacuum packaging LCVG#01 and the control circuit was adapted to maintain the resonator in a stable four-wave belly vibration mode and extract the detected signal to obtain the angular rate [17]. In the process, the force to rebalance (FTR) control was used to suppress the secondary mode vibration, which would improve the working bandwidth, linearity, and detection sensitivity of the gyroscope [18,19,20].

In the experiment of piezoelectric excitation and detection, it was found that the detection signal was very weak and doped with a low-frequency interference signal. Therefore, the filter amplifier circuit was designed. The detection signal terminal through the filter amplifier circuit was connected to the data acquisition card. The voltage signal was generated by the LabVIEW open-loop test system to excite the vibration, and the detection signal was demodulated and analyzed to calculate the vibration characteristic parameters.

#### 4.3.1. Frequency Sweep Test of the Resonator Based on LabVIEW

**Figure 12:**
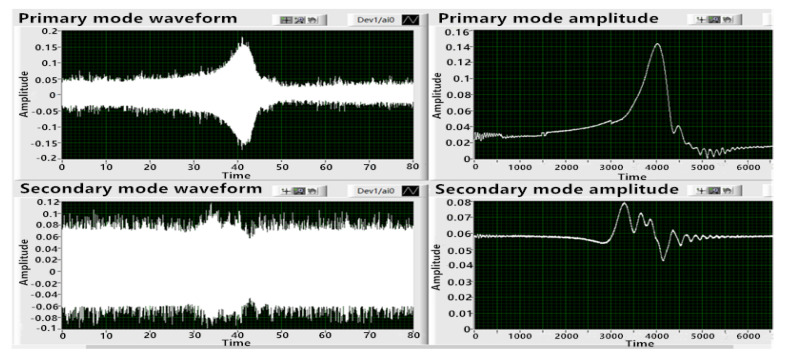
Frequency sweep results of two modes when electrode pair 1 was excited.

Similar to the piezoelectric test, the first step was frequency sweep. It was known that the eigenfrequency of LCVG#01 was about 7758 Hz, so 7758 Hz was set as the center frequency of the sweep, the sweep span was 4 Hz, the step size was 0.05 Hz, and the sweep time was 80 s. Firstly, the electrode pair 1 was used as the excitation electrode, the electrode pair 3 was used as the primary mode detection electrode, and the electrode pair 2 was used as the secondary mode detection electrode. The results are shown in Figure 12.

**Figure 13:**
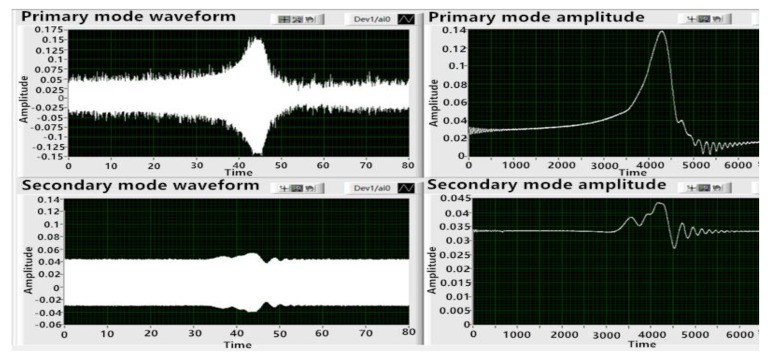
Frequency sweep results of two modes when electrode pair 3 was excited.

When electrode pair 1 was excited, the waveforms of the primary mode and the secondary mode were consistent with the expectation. However, the signal value of the secondary mode was too large compared with that of the primary mode, and the local value was even larger than that of the primary mode. Therefore, electrode pair 3 was used as the excitation electrode, electrode pair 1 was used as the primary mode detection electrode, and electrode pair 4 was used as the secondary mode detection electrode. The sweep test was repeated. The results are shown in Figure 13.

It can be seen from Figure 13 that the sweep envelope curve obtained by exciting electrode pair 3 was smoother, the signal noise was smaller, and the frequency peak values of the sweep signal of the primary mode and the secondary mode were more reasonable, which was conducive to the control of the circuit. After that, the frequency sweep was carried out with electrode pair 2 and electrode pair 4 as the excitation electrodes, successively, which failed to realize normal excitation. Therefore, it was determined that electrode pair 3 was used as the excitation electrode, electrode pair 1 was the primary mode detection electrode, and electrode pair 4 was the secondary mode detection electrode.

#### 4.3.2. Phase-Locked, Amplitude Stabilized, and Single-Frequency Excitation Experiments

When electrode pair 3 was used as the excitation electrode, the eigenfrequency was 7759.39 Hz by the frequency sweep. The resonator was excited by the signal with the eigenfrequency. The phase locked loop (PLL) and amplitude gain control (AGC) are essential control loops for stabilizing the resonator’s vibratory frequency and amplitude. The PLL and AGC modules were achieved in LabVIEW with its inner PID (Proportion Integral Derivative) controller, low-pass filter, and so on. The PID control parameters of PLL and AGC were adjusted continuously and carefully. Finally, the appropriate control parameters were determined.

**Figure 14:**
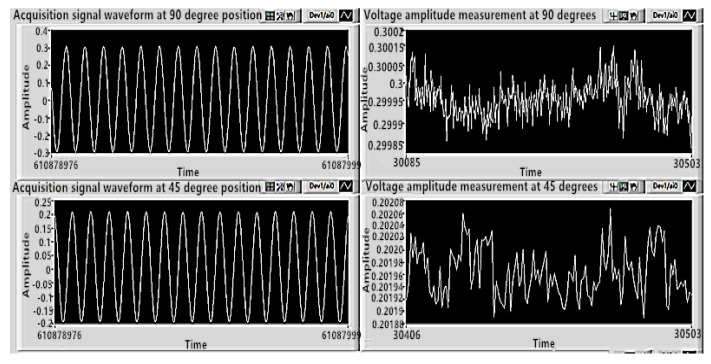
The primary mode detection voltage and the secondary mode detection voltage when the resonator vibrates stably in the form of eigenmode.

When the voltage amplitude of the excitation signal was set to 7.5 V, the primary mode detection voltage of electrode pair 1 was about 0.3 V, and the secondary mode detection voltage of electrode pair 4 was about 0.202 V. Detailed results are shown in Figure 14, the amplitudes of the primary mode detection voltage and the secondary mode detection voltage were stable in the thousandth place, and the stability effect was good.

**Figure 15:**
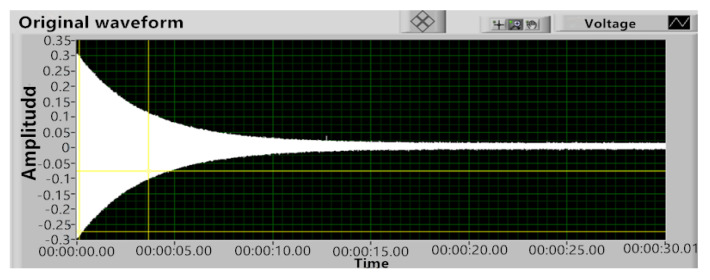
Free decay curve of the resonator vibration.

Under the condition that the CQR vibrated steadily in the four-wave belly vibration mode, the excitation signal was interrupted to make its vibration decay freely, so that the decay time was recorded and the Q factor was calculated. Figure 15 shows the free attenuation curve after the interruption of the excitation signal, in which attenuation time τ was 3.41 s and the Q factor was 8.31 × 10^4^, the Q factor was a little lower than that in Table 2, because of the measurement error and pressure difference.

### 4.4. Closed-Loop Gyroscope Performance Test

**Figure 16:**
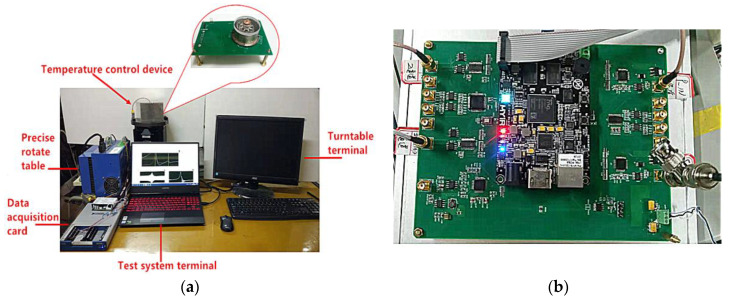
(**a**) Closed-loop gyro effect test system; (**b**) gyro measurement and control circuit.

Through the open-loop test, the appropriate excitation and detection electrodes, phase-locked loop parameters, and excitation signal were determined. Using the test system shown in Figure 16a, the gyroscope was installed into the temperature control device, the temperature control device was fixed on the turntable, and the closed-loop gyroscope performance test was conducted. Figure 16b shows the measurement and control circuit of the gyroscope.

#### 4.4.1. Scale Factor Test

Scale factor refers to the ratio of the output angular velocity to the input angular velocity. In the actual test, a series of numerical relations between the output angular velocity and the input angular velocity were fitted, and the fitting slope obtained was taken as the scale factor, namely:

(1) $$S_{\Omega }=\frac{dy}{dx}$$

where *x* and *y* are input angular velocity and output angular velocity, respectively.

**Figure 17:**
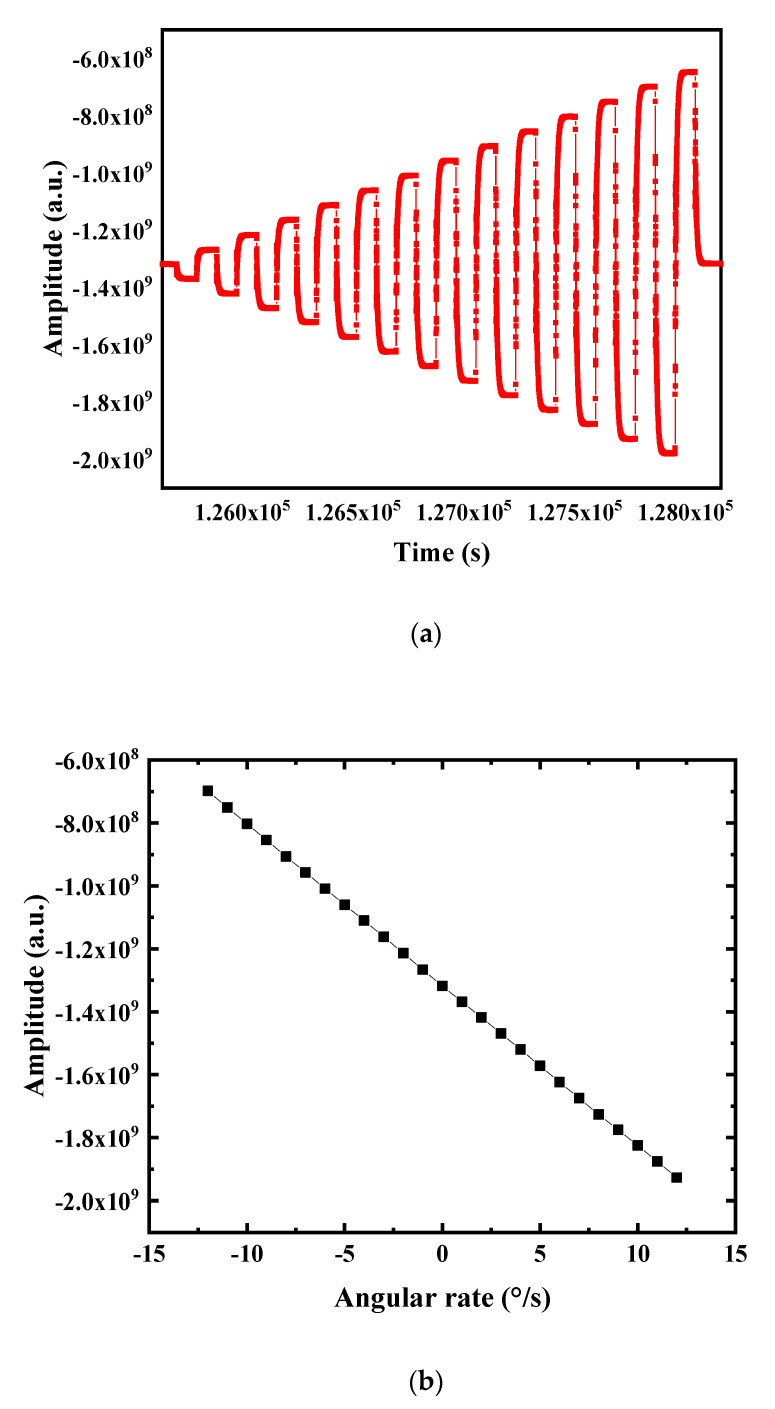
(**a**) Scale factor test raw data; (**b**) scale factor test fitting chart.

Power was supplied to the gyroscope control circuit. When working stably, the rotation speed of the turntable was set to change from 0°/s to ±20°/s with a gradient of 1°/s. The data acquisition time at each rotation speed was 90 s. It was found that when the external angular velocity was greater than 15°/s, the linear relationship between the output and the input was distorted, and the scale factor had a large error. Therefore, considering the data fitting within ±12°/s, the amplitude of the original output signal changed with time, as shown in Figure 17a.

The stable signal output value at each angular velocity was taken as the corresponding signal output at this angular velocity, and a graph drawn to get its corresponding relationship and conduct linear fitting, and get the relationship between them, as shown in Figure 17b. The fitting linear expression is:

(2) y=−51170384.6153846x−1315292000

In the range of ±12°/s angular velocity, the scale factor = −51170384.6153846, R^2^ = 0.99998510, and the linearity of the scale factor is good.

#### 4.4.2. Bias Test

Bias refers to the discrete degree of the output signal of the gyroscope in the state of zero input, which is expressed by the mean value of the long time output converted into the input angular velocity. The long-time steady-state output will fluctuate around the mean value when the input angular speed is zero.

**Figure 18:**
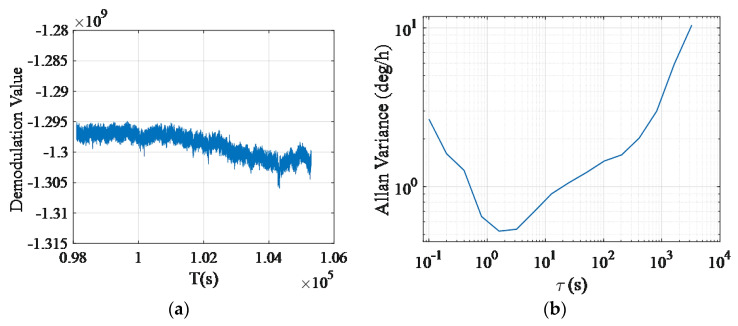
(**a**) 2-h bias raw data; (**b**) Allan variance curve corresponding to the 2-h bias.

Under the condition of no angular velocity input, the gyroscope was made to run stably. The relatively stable bias data were taken at a sampling rate of 10 Hz within two hours, and the results are shown in Figure 18a; the *x* axis is the time, and the *y* axis is the demodulation value with arbitrary unit. The bias stability and Angular Random Walk (ARW) were calculated with the normalized Allan Variance method with the following formula:

(3) $$\sigma \left(\tau_{M}\right)=\sum_{n=-2}^{2}A_{n}{\left(\tau_{M}\right)}^{\frac{n}{2}},$$

where *σ*^2^(*τ_M_*) is the Allan Variance with *M* data as a group, *A*_n_ is the model coefficient, *n* = −2, −1, 0, 1, 2. Bias stability is equal to *A*_0_, Angular Random Walk (*ARW*) is calculated as:

(4) $$ARW=\frac{A_{-1}}{60}.$$

At this time, the corresponding bias stability was about 0.790°/h, and the Angular Random Walk (*ARW*) was about 0.012°/√h, as shown in Figure 18b.

## 5. Discussion

The test results before and after sintering were compared by using the acoustic signal as the excitation source. After sintering, the Q factor changed most obviously, the Q factor of HCVG#01 decreased by 50%, and that of LCVG#01 decreased by 35%. This shows that the sticking and sintering of the PCEs have an impact on the CQR. The frequency split of HCVG#01 increased, while that of LCVG#01 decreased. This shows that the introduction of the PCEs has a slight effect on the symmetry of resonators.

By comparing the piezoelectric excitation results after polarization with the acoustic excitation results after sintering, we found that the Q factor of LCVG#01 resonator decreased, the frequency split slightly increased, and the overall quality of the resonator decreased. This phenomenon may be caused by the error introduced by the welding wire. The piezoelectric detection voltages of LCVG#01 were only 10–20 mv, which is smaller than the simulation results. It is speculated that the reason may be that the polarization is not sufficient.

In the experiment of piezoelectric excitation and detection of HCVG#01, the Q value was only 2 × 10^4^, and the frequency splitting was 1.17 Hz. Compared with the results of the acoustic excitation experiment after sintering, the Q value suffered a precipice fall, and the frequency split was nearly doubled. It is indicated that the fracture of the electrode has a great influence on the structural symmetry and mass uniformity of the resonator.

Through the analysis of gyroscope performance parameters, it was found that the high-temperature sintering scheme is feasible, but the gyroscope precision was not very high. In view of this problem, we speculate the following reasons and propose improvement methods: First, the circle holes at the bottom of the CQR were too large, which limited the position of the PCEs, so that the PCE position did not coincide with the primary axis, thus affecting the precision. On this issue, the size and position of the circle holes can be optimized to let the position of the PCEs coincide with the primary axis. Second, for the LCVG#01, the welding spot obtained by common soldering method was too large, which would have a negative impact on the quality of the resonator. Therefore, we can optimize the resonator to get a more suitable packaging structure and introduce spot welding. Third, the polarization of the PCEs may not be sufficient. In this regard, we can make a detailed comparative excitation experiment between the polarized PCEs and the initial PCEs to determine the polarization efficiency, and learn from the conclusion in the field of piezoelectric ceramic production to explore the most appropriate polarization conditions. Fourth, the improper assembly resulted in the increase of vibration loss and eccentricity error of resonator, which restricted the improvement of gyroscope precision. For this reason, we can use precision machining and matching design to process the special resonator assembly workpiece, so as to ensure that the assembly will not introduce or minimize the introduction of new errors. Finally, the existing control circuit was not perfect, and the circuit noise may be too large. On this issue, the existing control circuit can be improved to reduce the circuit noise and optimize the amplifier compensation circuit to improve the gyroscope precision.

## 6. Conclusions

Because of the disadvantages of the existing epoxy bonding method, a new high-temperature sintering method was proposed, and the corresponding electrode positioning fixture and printing screen were designed to sinter the CQR and the PCEs together. In the whole process, the quality of the CQR was relatively good. The LCVG#01 was packaged and adapted to the circuit, then the gyroscope was obtained. The open-loop test was carried out to determine the excitation and detection conditions and phase-locked loop parameters for the closed-loop test. The closed-loop gyroscope performance test was carried out, and the evaluation parameters such as the scale factor, bias stability, and ARW were obtained. The gyroscope effect was seen and a series of tests were made on the gyroscope precision. Although the precision of the gyroscope prototype is limited, the test results preliminarily confirm the feasibility of the scheme. In order to develop a high-precision cylindrical quartz piezoelectric vibration gyroscope, further improvements are needed in the aspects of processing optimization of the resonator, precise positioning of electrodes, precise assembly of the resonator, circuit compensation and control, error analysis and processing.

## Figures and Tables

**Table 1:** Comparison of vibration parameters before and after sintering.

Resonator State	Parameter	HCVG#01	LCVG#01
Before sintering	Q factor	6.4 × 10^5^ (30.8 Pa)	3.4 × 10^5^ (29.97 Pa)
	Frequency split (Hz)	0.48	0.427
	Amplitude (nm)	4.16	3.56
	Rate (mm/s)	0.09098	0.10721
After sintering	Q factor	3.2 × 10^5^ (29.99 Pa)	2.2 × 10^5^ (29.5 Pa)
	Frequency split (Hz)	0.537	0.317
	Amplitude (nm)	3.85	3.53
	Rate (mm/s)	0.06259	0.04558
Q factor decrease rate		50%	35%

**Table 2:** Test results of vibration characteristics of LCVG #01 excited at different electrode pairs.

Excitation Electrode Pair	Q Factor	Frequency Split
1	147 k (0.67 Pa) 120 k (30 Pa)	0.415 Hz
2	114 k (0.24 Pa)	0.520 Hz
3	129 k (0.16 Pa)	0.482 Hz
4	92 k (0.13 Pa)	0.513 Hz

**Table 3:** Detection voltages of other electrode pairs under different excitation conditions.

	Detection	1	2	3	4
Excitation					
1			12.8 mV	17.4 mV	9.4 mV
2		12.1 mV		10.4 mV	15.2 mV
3		19.4 mV	9.44 mV		14.2 mV
4		9.8 mV	14.2 mV	13.2 mV	
